# Interferon-inducible chemokines reflect severity and progression in sarcoidosis

**DOI:** 10.1186/1465-9921-14-121

**Published:** 2013-11-07

**Authors:** Robert Su, Michelle-Linh T Nguyen, Misha R Agarwal, Christopher Kirby, Christine P Nguyen, Joris Ramstein, Eli P Darnell, Antonio D Gomez, Melissa Ho, Prescott G Woodruff, Laura L Koth

**Affiliations:** 1 Division of Rheumatology, University of California San Francisco, San Francisco, CA, USA; 2 Division of Pulmonary, Critical Care, Sleep and Allergy, University of California San Francisco, 505 Parnassus Avenue, Box 0111, San Francisco, CA 94143, USA; 3 Department of Medicine and Cardiovascular Research Institute, University of California San Francisco, San Francisco, CA, USA

**Keywords:** Sarcoidosis, CXCL10, CXCL9, Interferon inducible chemokines

## Abstract

**Background:**

Identification of serum proteins that track with disease course in sarcoidosis may have clinical and pathologic importance. We previously identified up-regulated transcripts for interferon-inducible chemokines CXCL9, and CXCL10, in blood of sarcoidosis patients compared to controls. The objective of this study was to determine whether proteins encoded by these transcripts were elevated in serum and identified patients with remitting vs. chronic progressive sarcoidosis longitudinally.

**Methods:**

Serum levels of CXCL9, CXCL10, and proteins associated with inflammation and/or disease activity (sIL2R, ACE, ESR and CRP) were measured in a prospective cohort of sarcoidosis subjects and controls. Comparisons were made between groups and clinical course using pulmonary function measures and a severity score developed by Wasfi *et al*.

**Results:**

In a cross-sectional analysis of 36 non-immunosuppressed sarcoidosis subjects, serum CXCL9, CXCL10, and sIL2R were significantly elevated compared to 46 controls (p < 0.0001). CXCL9 and CXCL10 were strongly inter-correlated (p = 0.0009). CXCL10 and CXCL9 were inversely correlated with FVC% predicted and DLCO% predicted, respectively. CXCL10 and CXCL9 significantly correlated with sarcoidosis severity score. sIL2R, ESR, CRP, and ACE serum levels did not correlate with pulmonary function measures or severity score. In the longitudinal analysis of 26 subjects, changes in serum CXCL10 level over time corresponded with progression versus remission of disease.

**Conclusions:**

Interferon-γ–inducible chemokines, CXCL9 and CXCL10, are elevated in sarcoidosis and inter-correlated with each other. Chemokine levels correlated with measures of disease severity. Serial measurements of CXCL10 corresponded to clinical course.

## Background

Sarcoidosis is a systemic inflammatory disease characterized by the presence of noncaseating granulomas that predominantly affect the lungs. The natural course of sarcoidosis is heterogeneous; the majority of patients have a self-limiting course, but one-third of patients develop progressive disease [1,2]. Our prior study revealed a robust peripheral blood gene expression signature in sarcoidosis with an over-representation of genes related to interferon (IFN) Type I and II signaling pathways [3]. Notably, many of these IFN pathway-related genes were also upregulated in lung tissue from sarcoidosis subjects compared to controls, such as downstream signaling molecules (e.g. *IRF1*), which correlated with the severity of lung disease, suggesting a concordance of gene regulation in both blood and lung tissue compartments [3].

In addition to upregulation of genes downstream of IFN signaling pathways, we also found concordance in the blood and lung of increased transcript levels for several chemokine genes that are directly induced by IFN-γ [3]. These genes include CXCL9 (aka, Monokine induced by IFN-γ or MIG) and CXCL10 (aka, IFN-γ-inducible protein, 10 kDa or IP-10). Prior experimental studies have shown that a major source of these chemokines are monocytic cells after IFN-γ stimulation [4,5]. These chemokines signal through the receptor, CXCR3, which is an inflammatory receptor found on CD4+ Th-1 and CD8+ cytotoxic T cells [6]. Additional evidence suggests that CXCR3 and its ligands promote recruitment of T cells into inflamed peripheral tissue in human autoimmune diseases [7]–[9]. In sarcoidosis, several studies have identified elevations of these chemokines in bronchoalveolar lavage fluid (BALF) [10]–[13], suggesting they may contribute to lung inflammation. Therefore, since transcripts for these chemokines are upregulated in lung tissue ([14], GSE16538), and the proteins themselves were shown to be elevated in BALF, we hypothesized that blood levels of these chemokines could reflect disease severity and clinical course in sarcoidosis. We tested this hypothesis in a well-characterized prospective cohort of sarcoidosis and control subjects. Understanding how longitudinal assessment of these circulating chemokines relate to disease course may lead to new non-invasive ways to predict which patients will have persistent and/or progressive disease and to determine which patients may be suitable for therapies which target interferons.

## Methods

### Study population and clinical data

We collected blood samples and clinical data prospectively from a longitudinal cohort of sarcoidosis subjects and healthy controls at University of California San Francisco (UCSF) recruited from the community at large. Exclusion criteria included a medical history of chronic infection, tuberculosis, autoimmune diseases, cancer, diabetes or any condition associated with immune dysregulation. All sarcoidosis subjects met diagnostic criteria based upon established ATS guidelines [15]. Duration of disease was defined as time since date of biopsy confirmation of sarcoidosis. All sarcoidosis subjects had spirometry values within 6 months of blood draws [16]. Spirometry (nSpire HDpft 1000, Longmont, CO, USA) was performed at each subsequent follow-up visit using ATS guidelines [17]. The study was approved by the Committee on Human Research at UCSF (IRB 10–03759).

For the cross-sectional analysis, only subjects who had at least one baseline serum sample and were not on immunosuppressive therapy within 3 months of the blood draw were included to avoid any unknown and potential effects of immunosuppression on serum protein levels. (See Additional file 1 for patient selection flowchart).

In contrast, the longitudinal analysis included sarcoidosis patients with at least *two* serum samples collected at 6 to 12 month intervals, regardless of whether or not they had received immunosuppressive therapy within 3 months of the blood draw (see Additional file 1). Based on prior studies [18]–[21], subjects in the longitudinal analysis were classified into one of two groups: chronic versus remitting. Chronic sarcoidosis subjects were defined as individuals who required systemic immunosuppression for recurrent or ongoing disease activity 2 years after their initial diagnosis. Remitting sarcoidosis subjects were defined as patients who either A) did not require systemic immunosuppression beyond 2 years after diagnosis or B) never required immunosuppression ever since diagnosis.

Levels of routine inflammatory markers were obtained for all subjects. Serum C-reactive protein (CRP) level and erythrocyte sedimentation rate (ESR) testing was performed by the UCSF CLIA-approved clinical laboratory. Angiotensin converting enzyme (ACE) level was measured by Quest Diagnostics Nichols Institute (San Juan Capistrano, CA, USA). All sarcoidosis subjects were radiographically classified using Scadding stage and assigned a clinical sarcoidosis severity score developed and published by Wasfi et al. [22]. The Wasfi score is a model built using backward regression multivariate analysis to identify variables reflective of sarcoidosis severity, relying on clinical assessment scores by a panel of independent sarcoidosis experts at National Jewish Medical and Research Center as the “gold standard” for determining disease severity. We recognize that a major limitation to this model is that there is no universally-recognized “gold standard” in evaluating sarcoidosis severity (a motivating factor in the design of our study) but this severity scoring model was selected for the purposes of our study because the model had been validated by a separate international panel of sarcoidosis experts, incorporates extrapulmonary sites of involvement, and utilizes clinical variables that are easily accessible to clinicians at a single patient visit. The severity score is calculated as follows:

Severity Score = 11.46 + 3.9(Cardiac) + 2.56(Neuro) + 1.56(Immunosuppression within 30 days) – 0.051(FVC% predicted) +1.75(African American) – 0.054(FEV1/FVC ratio)

where Cardiac = 1 if there is cardiac involvement, 0 if not; Neuro = 1 if there is neurologic involvement, 0 if not; Immunosuppression = 1 if receiving noncorticosteroid immunosuppression therapy, 0 if not; African American = 1 if the subject was African American, 0 if not; FVC = forced vital capacity; FEV1 = forced expiratory volume in 1 second.

### Sample processing

Blood samples were collected in 10-ml Vacutainer red/gray tubes (BD, Franklin Lakes, NJ, USA). Samples were processed for serum after incubation at room temperature for 30 minutes and then centrifugation at 2400 rpm for 12 minutes. Samples were aliquoted and stored at −80°C until thawed for Luminex assays.

### Multiplex protein assay

Multiplex bead-based assay kits for CXCL9, CXCL10, and sIL2R were purchased from Luminex Corporation (Austin, TX, USA). The multiplex assay also measured IL6, IL2, IL15, TNF-α, and CCL3; however, the concentrations of these analytes were below the lower limit of quantitation and thus excluded from further studies. The lowest detectable levels for sIL2R, CXCL10, and CXCL9 were 29.4 pg/ml, 1.6 pg/ml, and 12.3 pg/ml, respectively. Nine standard control samples were analyzed to ensure validity of results. All samples were analyzed in duplicate, and the averaged values were used for each sample. Measurements with a coefficient of variation less than 0.2 between duplicate samples were included for data analysis.

### Statistical analysis

Fisher’s exact test was used to compare nominal clinical characteristics between sarcoidosis and control subjects. Distribution of sIL2R, CXCL9, and CXCL10 levels were assessed for normality and log10 transformation was applied for non-parametric data. In some instances, the distribution of data remained non-parametric even after transformation. Therefore, unpaired two-tailed t-test or the Wilcoxon rank sum statistic, as appropriate, was used to compare differences between groups. A non-parametric test for trend was performed in Stata version 12 (StataCorp, College Station, TX, USA) in sensitivity analyses to assess the effects of immunosuppression [23]. Pearson’s correlation coefficient or Spearman’s rank correlation coefficient was used, as appropriate, to evaluate the relationships between CXCL9 and CXCL10 and pulmonary function measurements and severity score in sarcoidosis subjects. Cross-sectional analyses were performed in JMP version 10 (SAS, Cary, NC, USA). P < 0.05 was considered statistically significant.

## Results

### Cross-sectional study patient characteristics and baseline blood measurements

The clinical characteristics of the cross-sectional study population are summarized in Table 1 and Additional file 2. Despite the mean age of the sarcoidosis group being older than the control group, there were no statistically significant relationships between CXCL9, CXCL10, and sIL2R levels and age at blood draw or duration of disease (p > 0.5). Gender, ethnicity and smoking status were similar between the two groups. All subjects in this cross-sectional analysis had pulmonary sarcoidosis. In addition, 17 also had extrapulmonary involvement (1 subject had cardiac sarcoidosis, 9 subjects had ocular sarcoidosis, 7 subjects had central nervous system sarcoidosis). Sarcoidosis subjects were lymphopenic compared to control subjects (Table 2). The concentrations of ACE, CRP, ESR, CXL9, CXCL10, and sIL2R were all significantly elevated in sarcoidosis subjects when compared to control subjects (Table 2).

**Table 1:** Cross-sectional cohort characteristics

	**Control**	**Sarcoidosis**	**P-Value**
	**N = 46 (%)**	**N = 36 (%)**	
Female	28 (61%)	23 (64%)	0.82
Age (y)^*^	42.15 ± 12.96	52.06 ± 11.57	<0.01
Race			
African-American	5 (11%)	2 (5%)	0.46
White	33 (72%)	33 (92%)	0.02
Other	8 (17%)	1 (3%)	0.50
Ethnicity			
Hispanic	6 (13%)	2 (5%)	0.46
Smoking Status			
Ever	11 (24%)	14 (39%)	0.16
Current	4 (8.7%)	1 (3%)	0.38
Cardiac/ocular/CNS	n/a	1/9/7	
Pulmonary Function			Range
FEV1 (%)^*^		93.9 ± 17.5	47-133
FVC (%)^*^		100.0 ± 13.8	63-129
FEV1/FVC (%)^*^		72.9 ± 8.4	56.2-94.8
TLC (%)^*^ N = 25		99.9 ± 15.0	62-124
DLCO (%)^*^ N = 27		87.77 ± 15.7	45-120
Immunosuppression	None	No immunosuppression at time of or within 3 months of blood draw	

*Data are presented as mean ± standard deviation.

**Table 2:** Blood and Serum Measurements*

**Group**	**Lymphocytes (x10**^**9**^**/L)**	**ACE (U/L)**	**CRP (mg/L)**	**ESR (mm/h)**	**CXCL9 (pg/ml)**	**CXCL10 (pg/ml)**	**sIL-2R (pg/ml)**
Control	1.68	36.5	1.6	5	29.4	15.53	12.38
	(1.1-3.1)	(0–101)	(0–24.5)	(0–30)	(29.4-73.3)	(6.6-25.0)	(12.3-58.6)
	N = 46	N = 46	N = 46	N = 44	N = 34	N = 46	N = 46
Sarcoidosis	1.26†	50‡	2.8¥	9¥	56.73†	28.72†	40.84†
	(0.6-2.1)	(0–147)	(0.9-18.3)	(0–55)	(29.4-165.9)	(13.3-80.1)	(12.3-158.6)
	N = 35	N = 35	N = 35	N = 34	N = 30	N = 36	N = 36

*Data are presented as median (range); sample size per measurement is indicated.

†P < 0.0001 comparing control to sarcoidosis.

¥P < 0.001 comparing control to sarcoidosis.

‡P < 0.0253 comparing control to sarcoidosis.

### Sarcoidosis and control serum protein comparisons

**Figure 1:**
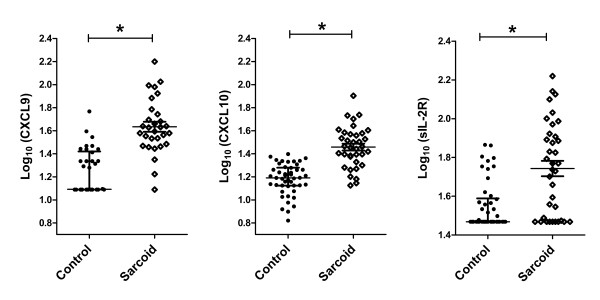
**Comparison of serum CXCL9 (N = 30), CXCL10 (N = 36), and sIL-2R (N = 36) protein levels in non-immunosuppressed sarcoidosis subjects.** Data are presented as median and interquartile range. *p < 0.0001.

**Figure 2:**
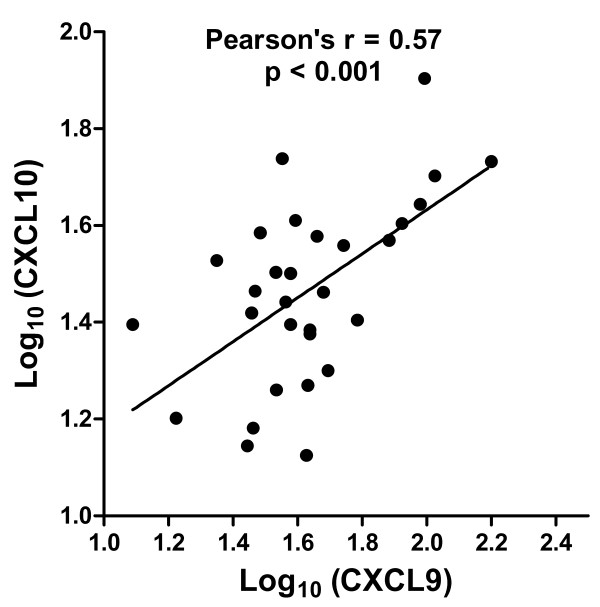
Serum levels of CXCL10 and CXCL9 in non-immunosuppressed subjects with sarcoidosis (N = 30).

We found statistically significant increases in CXCL9, CXCL10, and sIL2R in sarcoidosis subjects compared to control subjects (Figure 1). We also found significant inter-correlation between the two chemokines within each subject (Figure 2). CXCL9 was weakly inter-correlated with sIL2R (Spearman’s ρ = 0.3764, p = 0.04), while CXCL10 was not (Spearman’s ρ = 0.0931, p = 0.59).

### Correlations with disease severity

**Figure 3:**
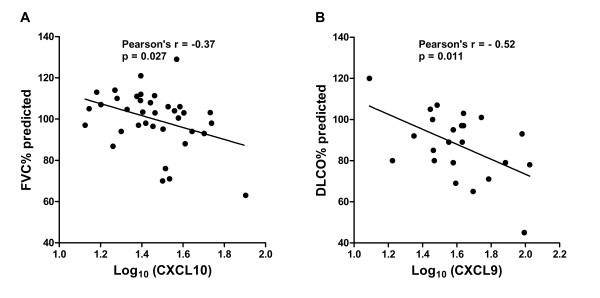
**Relationship between interferon-inducible chemokines and pulmonary function testing. (A)** Serum CXCL10 is inversely correlated with FVC% predicted and (N = 36) **(B)** serum CXCL9 is inversely correlated with DLCO% (N = 23) predicted in non-immunosuppressed subjects with sarcoidosis.

**Figure 4:**
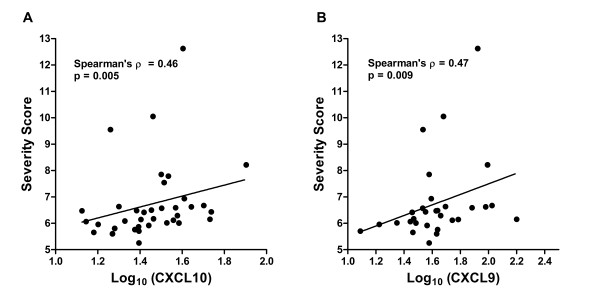
**Relationship between interferon-inducible chemokines and clinical severity. (A)** Serum CXCL10 (N = 36) and **(B)** CXCL9 (N = 30) positively correlated with a sarcoidosis severity score previously published by Wasfi *et al*.

There were significant inverse correlations between CXCL10 and FVC% predicted (Figure 3A) and CXCL9 and DLCO (diffusing capacity) % predicted (Figure 3B). There was a trend for CXCL10 and DLCO% predicted (Pearson’s R = −0.37, p = 0.056). As an alternative method to examine how these chemokines related to disease severity, we used a severity score developed by Wafsi *et al.* which incorporates lung function measures, presence of cardiac and/or neurologic disease, and immunosuppression use [22]. Using this equation, both CXCL10 and CXCL9 were significantly correlated with severity score (Figure 4). Of note, levels of non-specific markers of inflammation as well as “sarcoidosis-related” serum markers, such as sIL2R, ESR, CRP, and ACE level, did not correlate with pulmonary function measures or severity score.

### Correlations with disease progression

**Figure 5:**
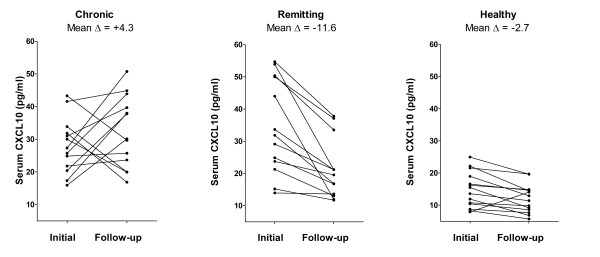
**Changes in serum CXCL10 levels correspond to sarcoidosis disease course.** CXCL10 remains persistently elevated in patients with chronic sarcoidosis (N = 13). CXCL10 declines over time in patients with remitting sarcoidosis (N = 13). Longitudinal serum CXCL10 levels in 14 healthy controls are presented for comparison.

Twenty-six subjects with sarcoidosis had longitudinal data (Table 3), meaning they had at least 2 serum samples. In the longitudinal measurements, the majority of subjects with chronic sarcoidosis demonstrated persistently elevated or increased serum CXCL10 levels, whereas remitting sarcoidosis subjects consistently showed a decrease in serum CXCL10 (Figure 5). The mean increase in serum CXCL10 in chronic sarcoidosis was 4.3 ± 14 pg/ml, compared to a drop of 12.8 ± 11 pg/ml in the remitting sarcoidosis group, p = 0.002 (Figure 5). Patients with chronic sarcoidosis demonstrated a mean decline in FVC of 0.13 L ± 0.13 L, whereas remitting subjects demonstrated a mean improvement in FVC of 0.11 ± 0.34 L (p = 0.051). There was no difference in time interval between performing pulmonary function testing between both groups (10.3 ± 5.3 versus 8.9 ± 5.2 months, respectively, p = 0.53).

**Table 3:** Clinical characteristics of longitudinal cohort by disease progression

**Total N = 26**	**Chronic**	**Remitting**
No. subjects	13	13
Female	9 (69%)	8 (62%)
Age*	53.3 ± 12	54.3 ± 11.5
Ethnicity (White/Black/Other)	10/3/0	12/0/1
Time from diagnosis to baseline blood draw (years)*	8.4 ± 10.1	1.6 ± 1.1
Scadding stage at enrollment (I/II/III/IV)	0/5/3/5	3/7/1/2
Immunosuppression regimen history** (no. subjects)	4 prednisone, 1 azathioprine, 5 methotrexate + prednisone, 2 methotrexate + prednisone + plaquenil, 1 prednisone + plaquenil	1 prednisone, 1 methotrexate + prednisone, 11 never treated
Number of subjects receiving immunosuppression at time of baseline blood draw or follow-up blood draw	8	0
Δ in serum CXCL10 (pg/ml)*	+4.3 ± 14	-11.6 ± 11
Interval between baseline and follow-up visits (months)*	6.47 ± 1.55	6.90 ± 1.89
Δ in FVC (L)*	-0.13 ± 0.13	+0.11 ± 0.34

*Data expressed as mean ± SD.

**Systemic immunosuppression history includes medications that were ever taken for sarcoidosis, and not necessarily medications taken at time of blood draws.

**Figure 6:**
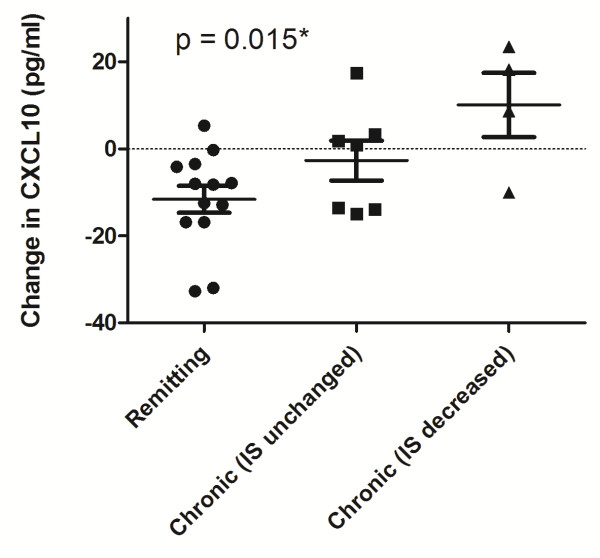
**The effect of systemic immunosuppression (IS) on serum CXCL10.** In remitting disease subjects who did not receive IS at baseline and followup (N = 13), CXCL10 declined spontaneously. In chronic disease, CXCL10 remained largely unchanged if immunosuppression was unchanged (N = 7), and increased if immunosuppression was decreased (N = 4). *denotes a statistically significant result using the non-parametric trend test.

To determine whether changes in immunosuppression influenced change in CXCL10 levels in this longitudinal analysis, we performed a sensitivity analyses. We defined three groups of subjects based on immunosuppression use: 1) remitting disease (not on immunosuppression at any time), 2) chronic disease patients with no change in immunosuppression (N =7) and 3) chronic disease patients in whom systemic immunosuppression was decreased in dose over time (N = 4). No subject had an increase in immunosuppression and 2 subjects were excluded from the sensitivity analysis because the dosages of immunosuppressive therapy were missing. We found that CXCL10 levels decreased in remitters (−11.6 ± 11 pg/ml), showed little change in chronic disease when immunosuppression was unchanged (−2.7 ± 12 pg/ml), and increased in chronic disease when immunosuppression was decreased (+10.1 ± 15 pg/ml) (p = 0.015 for trend test, Figure 6). These data suggest that CXCL10 levels may reflect disease activity, decreasing in remitting patients, remaining elevated in those with chronic disease, and increasing further when immunosuppression is decreased in the presence of chronic disease.

Subjects with chronic sarcoidosis were also likely to show persistent elevations in CXCL9 compared to those with remitting sarcoidosis; however, the difference in change of CXCL9 over time between these groups did not reach statistical significance (+7.22 pg/ml versus −5.19 pg/ml in chronic and remitting groups, respectively; p = 0.37). Changes in ACE, CRP, ESR, and sIL2R at follow-up did not correlate with disease progression (data not shown).

## Discussion

Based on our prior data showing upregulation of many genes related to Type I and II interferon signaling pathways in the blood, as well as clinical studies measuring elevated levels of IFN-γ-inducible chemokines in BALF [10]–[13], we hypothesized that circulating blood levels of the IFN-γ-inducible chemokines, CXCL9 and CXCL10, would reflect disease severity and clinical course over time. We found that CXCL9 and CXCL10 were indeed elevated in the serum of sarcoidosis subjects compared to controls and further, found that their levels correlated with the lung function measurements DLCO and FVC, respectively. In addition, we found that both chemokines correlated with a clinical severity score, which takes into account not only lung function measures, but also involvement of organs such as cardiac and the nervous system [22]. In the longitudinal analysis, subjects with chronic sarcoidosis were significantly more likely to show persistent elevations in CXCL10, whereas those with remitting sarcoidosis had declines in CXCL10 over time.

Although both CXCL9 and CXCL10 are IFN-γ-inducible proteins, our results suggest that changes in CXCL10 may play a larger role in, or track more closely to, disease progression compared to CXCL9. Although CXCL9 levels were correlated with CXCL10 levels and the direction of change in CXCL9 trended with direction of disease course, the difference in change of CXCL9 at follow-up between the chronic and remitting groups did not reach statistical significance. We speculate that the difference between CXCL9 and CXCL10 serum levels may, in part, be explained by the fact that CXCL10 production is also strongly induced by Type I interferons (α/β), which is not the case for CXCL9 [5,24]. Thus, individual sarcoidosis patients with higher circulating levels of CXCL10 compared to CXCL9 may have greater activation of the Type I and II interferon pathways, which we speculate could identify subsets of sarcoidosis phenotypes. In support of this idea, Antoniou *et al*. found a 12-fold elevation of circulating CXCL10 but non-significant elevation CXCL9 in sarcoidosis subjects compared to controls [25]. Similarly, our prior whole blood gene expression data from sarcoidosis and control subjects confirmed that transcripts for CXCL10 were significantly more upregulated compared to CXCL9 in sarcoidosis (Gene Expression Omnibus, GSE19314).

Notably CXCL10 is strongly expressed in sarcoidosis tissues [10]. Furthermore, a number of studies have measured CXCL10 in BALF from sarcoidosis patients [10]–[13,26]. CXCL10 has been shown to play a significant role in granulomatous inflammation. In mice models of granulomatous disease induced by *Propionobacterium acnes*, CXCL10 has been shown to be necessary for the formation of Th1 lymphocyte clusters in draining lymph nodes [27], and blockade of CXCR3 (the receptor for CXCL9 and CXCL10) and CCR5, inhibits the formation of granulomas in lungs [28]. In models of hypersensitivity pneumonitis, CXCL10 has also been shown to be necessary for granuloma formation [29,30]. Induction of CXCR3 with CXCL9 and/or CXCL10 allows for T cell migration into organ tissues, including those that are typically thought of as immune privileged, and contributes to Th1 cell differentiation, which are common features of sarcoidosis [31,32]. Interestingly, serial measurements of serum CXCL10 has been correlated with persistence or resolution of autoimmune thyroid disease [33], which is seen frequently with sarcoidosis [34]. Taken together, these findings could suggest that CXCL10 may contribute to Th1 cell recruitment and promote granulomatous inflammation in chronic sarcoidosis.

We also found that CXCL10 remained elevated among chronic sarcoidosis subjects compared to healthy controls despite immunosuppression use. Since use of immunosuppression can both reflect disease activity and potentially affect CXCL10 levels, we performed a sensitivity analysis, classifying patients with chronic disease according to changes in immunosuppression levels over time. This analysis revealed that CXCL10 levels remain elevated in patients classified as having chronic disease when immunosuppression is unchanged, but that CXCL10 levels increased further when immunosuppression was reduced.

With regard to serum sIL2R levels, we found significant elevations in patients with sarcoidosis which is consistent with prior data [35]–[38]; however, sIL2R levels failed to correlate with measures of disease activity or disease progression. This negative finding is compatible with the results of Grutters and colleagues, who showed that there were no differences in sIL2R between sarcoidosis subjects with or pulmonary function abnormalities [39]. Three other studies did not find any correlations in serum sIL2R with BAL fluid analyses [37,38,40]. Ziegenhagen *et al.* reported that an elevated serum sIL2R measured once at baseline was associated with progressive disease [36], which our and Grutters’ results do not support [39]. In contrast to our study, in the Ziegenhagen *et al.* cohort, the distinction between progressive and stable disease was made based on the patient’s disease course over a 6-month window after initial diagnosis, and these studies did not have follow-up measurements of sIL2R. Also, interpretation of sIL2R levels may be confounded by varying degrees of peripheral lymphopenia often observed in sarcoidosis independent of immunosuppression [1,41], since sIL2R is, in part, released by activated T lymphocytes [42]. Finally, sIL2R may be potentially affected by corticosteroid treatment in sarcoidosis [38,40]. Taken together, the characteristics of sIL2R may be the reason that it has not proven to reliably track with disease course.

There are important limitations to our study. Our cohort represented a single institution experience and although UCSF serves a multi-ethnic urban area, this study included predominantly white patients. Our sample size is limited and our findings warrant further study in larger validation cohorts. Although categorization of sarcoidosis subjects for clinical studies could be viewed as somewhat arbitrary, the goal in this study was to use a stratification system that had prior acceptance given the limitations of any categorization schema. The clinical classification scheme that we used to categorize sarcoidosis subjects as chronic vs. remitting was based upon previously published studies [18]–[21]. These studies defined chronic disease as sarcoidosis subjects who required systemic immunosuppression for recurrent or ongoing disease activity 2 years after their initial diagnosis. Remitting sarcoidosis subjects included patients who did not require systemic immunosuppression beyond 2 years after diagnosis, or never required immunosuppression. We could not apply the clinical categorizations of acute and non-acute sarcoidosis disease onset reported by Prasse *et al.*[43] since our cohort consisted of only non-acute disease.

## Conclusions

In summary, we found that serum CXCL9 and CXCL10 levels correlated with sarcoidosis disease severity as assessed by FVC and DLCO, as well as the Wasfi severity score, which incorporates additional clinical phenotypes of sarcoidosis. Changes in serum CXCL10 levels over time correlated with both chronic progressive disease (associated with sustained elevations of CXCL10) or remitting sarcoidosis (associated with a reduction over time in CXCL10). Our findings in the peripheral blood, along with data from several other cross-sectional BALF studies, suggest that CXCL9 and CXCL10 may play important roles in the pathogenesis of sarcoidosis. Larger replicate studies are needed to validate our findings.

## Abbreviations

sIL2R: Soluble interleukin-2 receptor; IFN: Interferon; ACE: Angiotensin converting enzyme; CRP: C-reactive protein; ESR: Erythrocyte sedimentation rate; UCSF: University of California San Francisco; ATS: American Thoracic Society; FVC: Forced vital capacity; FEV1: Forced expiratory volume in 1 second; DLCO: Diffusing capacity.

## Competing interests

The authors declare that they have no competing interests.

## Authors’ contributions

LK, RS, MLN, ADG, and PGW contributed to the study design, data acquisition and analysis, and writing of the manuscript. MA contributed to the data acquisition and statistical analyses. CK, CPN, JR, ED, and MH contributed to patient recruitment, study design, pulmonary function testing, blood collection, and data generation. All authors read and approved the final manuscript.

## Supplementary Material

Additional file 1 **Patient selection flow diagram for cross-sectional versus longitudinal cohorts.** This diagram depicts the selection criterion for sarcoidosis subjects in the cross-sectional and longitudinal analyses and, within the longitudinal cohort, the definitions of chronic versus remitting sarcoidosis. Click here for file

Additional file 2 **Characteristics of Cross-Sectional Sarcoidosis Subjects with Normal versus Low Pulmonary Function Testing.** This table compares the characteristics of cross-sectional sarcoidosis subjects with low pulmonary function testing (as defined by a FVC or DLCO of less than 80 percent predicted) with sarcoidosis subjects with normal pulmonary function testing. Click here for file
