# Supplementary material for: Interferon-inducible chemokines reflect severity and progression in sarcoidosis
Source: Respir Res. 2013 Nov 7;14(1):121. doi: 10.1186/1465-9921-14-121 (PMC4176097; doi:10.1186/1465-9921-14-121)
Supplement: Additional file 1 — Patient selection flow diagram for cross-sectional versus longitudinal cohorts. This diagram depicts the selection criterion for sarcoidosis subjects in the cross-sectional and longitudinal analyses and, within the longitudinal cohort, the definitions of chronic versus remitting sarcoidosis. [file 1465-9921-14-121-S1.docx]

**Additional File 1. Patient selection flow diagram for cross-sectional versus longitudinal cohorts.**

Sarcoidosis subjects with at least one serum marker sample (N = 56)

Subjects with immunosuppression use within 3 months of blood draw **excluded**.

Subjects with at least 2 serum markers, regardless of immunosuppression use.

Sarcoidosis **longitudinal** cohort (N = 26)

Sarcoidosis **cross-sectional** cohort (N= 36)

Required ongoing immunosuppression beyond 2 years of diagnosis.

1. Did not require immunosuppression beyond 2 years of diagnosis.

*Or*

1. Never required immunosuppression.

Chronic sarcoidosis

(N=13)

Remitting sarcoidosis

(N=13)
