# Supplementary material for: Interferon-inducible chemokines reflect severity and progression in sarcoidosis
Source: Respir Res. 2013 Nov 7;14(1):121. doi: 10.1186/1465-9921-14-121 (PMC4176097; doi:10.1186/1465-9921-14-121)
Supplement: Additional file 2 — Characteristics of Cross-Sectional Sarcoidosis Subjects with Normal versus Low Pulmonary Function Testing. This table compares the characteristics of cross-sectional sarcoidosis subjects with low pulmonary function testing (as defined by a FVC or DLCO of less than 80 percent predicted) with sarcoidosis subjects with normal pulmonary function testing. [file 1465-9921-14-121-S2.docx]

**Additional File 2.**  Characteristics of Cross-Sectional Sarcoidosis Subjects with Normal versus Low Pulmonary Function Testing

|  | Normal | Low (DLCO percent predicted or FVC percent predicted <80) | P value |
| --- | --- | --- | --- |
| N (Total = 36) | 27 | 9 |  |
| Age | 49.7 ± 11.1 | 59.2 ± 10.3 | 0.0326 |
| Male/Female | 10 / 17 | 3 / 6 |  |
| Ethnicity (White/Black/Other) | 24/1/2 | 8/1/0 |  |
| Extrapulmonary manifestations of sarcoidosis | 33% (n = 9) | 0 % (n=0) |  |
| Scadding stage 0/I/II/III/IV | 3/4/12/2/6 | 0/1/4/0/4 |  |
| Severity Score | 6.60 ±1.60 | 7.15 ± 0.71 | 0.1635 |
